# Supplementary material for: SyNDI: synchronous network data integration framework
Source: BMC Bioinformatics. 2018 Nov 6;19:403. doi: 10.1186/s12859-018-2426-5 (PMC6219086; doi:10.1186/s12859-018-2426-5)
Supplement: Supplementary file 5 — Files of the example presented in Additional file 4. This additional file is a zip package that contains all files used in Additional file 4. Detailed descriptions of each of these files are presented in a README file included in this zip package. (GZ 30411 kb) [file 12859_2018_2426_MOESM5_ESM.gz › SyNDITools/Meme2Fimo/meme_4.9.0_4.tar.gz!/meme_4.9.0/doc/shadow.xml]

shadow


# shadow

  
**Usage:**
> `shadow [options] <tree> <alignment>`

**Description:**

> Perform phylogenetic shadowing on a given DNA alignment, using a given
> tree (Boffelli et al, Science 2003). This program is a simplified
> version of `motiph`, in which the
> equilibrium distribution is set equal to the background model, rather
> than being taken from a given motif.

**Input:**

- `<tree>` is a phylogenetic tree in
  Phylip Newick format.
  This tree may contain additional species not represented in the alignment.
- `<alignment>` is a DNA multiple alignment in
  ClustalW format.

**Output:**
> Shadow will create a directory, named `shadow_out` by default.
> Any existing output files in the directory will be overwritten.
> The directory will contain:
>
> - An XML file named `shadow.xml` using the
>   CisML schema.
> - An HTML file named `shadow.html`
> - A plain text file named `shadow.text`
>
> The default can be overridden using the `--o` or `--oc`
> options which are described below.
>
> The `--text` will limit output to plain text sent to the standard output.

**Options:**


**Warning messages:** None
